# Geographical accessibility of cancer hospital using geospatial technology in Bagmati province, Nepal

**DOI:** 10.3389/fpubh.2026.1663989

**Published:** 2026-04-17

**Authors:** Basanta Kumar Neupane, Min Xu, Chunxiang Cao, Bipin Kumar Acharya, Yujie Yang, Shaohua Wang

**Affiliations:** 1 State Key Laboratory of Remote Sensing Science, Aerospace Information Research Institute, Chinese Academy of Sciences, Beijing, China; 2 University of Chinese Academy of Sciences, Beijing, China; 3 Planetary Health Research Center, Kathmandu, Nepal; 4 Nepal Open University, Lalitpur, Nepal

**Keywords:** accessibility, Bagmati province, cancer hospital, inequalities, travel scenario

## Abstract

**Background:**

Ensuring convenient access to cancer screening, diagnosis, and treatment is essential for mitigating and preventing the disease. Geographical accessibility plays a crucial role in cancer mitigation and healthcare planning, particularly in low-income countries such as Nepal. A spatial perspective is crucial for improving cancer mitigation planning and evidence-based policy formulation. This study examined the spatial accessibility and population coverage at the municipal level in Bagmati province, Nepal.

**Method:**

This study utilized spatial data, including LULC, DEM, OSM road networks, population distribution, and location of cancer hospitals, to calculate three different scenarios for reaching these hospitals. Based on that, a raster surface of travel time between residences and cancer hospitals was developed. We incorporated terrain, physical barriers, travel modes, speeds, and topography through various land-cover classes. Accordingly, underserved populations and zonal statistics were assessed using an interactive modeling approach to determine the spatial relationship between population, travel time, and zonal boundaries.

**Results:**

The motorized travel scenario exhibited the highest spatial accessibility, followed by the bicycling and walking scenarios. It varied significantly across the three scenarios. The motorized scenario has the highest accessibility, with 72.57% of people covered within a 30-min travel time. Motorized scenario has a maximum people coverage of 96.78% within a 360-min travel time. Kathmandu Valley and Chitawan urban areas showed higher accessibility across all scenarios, attributed to easy road networks. Rural and mountainous regions in the middle hills of the northern and eastern parts have low accessibility, attributed to steep slopes, scattered settlements, and sparse infrastructure.

**Conclusion:**

The findings demonstrate significant spatial disparities across various travel scenarios, underscoring the need for spatially informed health planning and policy development. These insights provide a foundation for addressing problems and enhancing cancer care delivery in Nepal using spatial techniques.

## 1 Background

Easy access to cancer hospitals for care and treatment is a fundamental component in achieving Universal Health Coverage (UHC), a key health policy mandate as the world advances toward the proposed United Nations’ Sustainable Development Goals (SDGs) (1). The geographical accessibility of a cancer hospital refers to the ease of access to a specific set of services offered by the hospital. Access can be defined as the opportunity to receive quality healthcare services in situations where there is a perceived need for care. Easy access is a basic need for patients. The principle of equal and equitable access is for those who need it, which is the foundation of the geographical accessibility approach (2). The Alma-Ata Declaration identified health care as the key point to achieving health for all, endorsed by the World Health Organization (3). At the 2018 Global Conference on Primary Health Care, all countries committed to achieving the UHC. To achieve the UHC without imposing financial burdens, it is essential to ensure equitable geospatial accessibility and to enhance geospatial justice (4, 5). To increase the health access of marginalized people, the global community, through the United Nations, has set seventeen SDGs and is targeting to be achieved by 2030 (6). Targeted goal 3, “Good health and well-being”, plays an essential role in promoting and connecting cancer patients to ensure access to cancer hospitals. This study aims to assess the geographical accessibility of cancer hospitals in Nepal and examine the spatial coverage using geospatial technology in Bagmati Province, thereby contributing one step toward progress toward SDG 3.

In recent decades, academia, health institutions, communities, and researchers in public health studies have paid considerable attention to geospatial technologies. They enhance the researcher’s capacity and increase the reliability of the research. Due to the substantial capacity to examine spatial components and relationships, geospatial technology is widely used to compute spatial disparity (7–9). Geospatial technologies are uniquely designed to assess topographic features and service center capacity. Previous spatial studies have uniquely examined accessibility analysis using AccessMod and ArcGIS (10), evaluating spatial disparities in health services. People have been using it to identify and solve several geographic problems; ultimately, it helps to reduce the spatial disparity between service providers and users (hospitals and patients) (11, 12). Several factors affect the examination of healthcare access, and summarized them using the 5A framework (acceptability, accessibility, affordability, accommodation, and availability). Besides 5A, geographic accessibility is one of the major factors of healthcare access. To analyze and explore spatial accessibility, the WHO suggests that travel time is a primary component, as it is more effective than distance (13). Previous analyses consider distance to be a major component in analyzing the accessibility and coverage capacity of service centers; they also used other components such as hospital locations, the capacity of the hospital, population distribution, topography, road networks, and geographical barriers (14–16).

Geographic accessibility refers to the ease with which people in a given area can access and utilize hospital services and facilities, which can be measured using various approaches (17). The study expressed in terms of the physical distance between the cancer hospitals and the user’s place of residence (18). Access to cancer hospitals is multidimensional. In this study, we use the conceptual framework described by Peters et al. The study focuses on two spatial dimensions, one examines geographical accessibility, and the other explores population coverage in Bagmati Province. Several factors influence the availability of health services. The inadequate number of doctors and medical staff, shortage of medicine and therapy services, lack of well-equipped hospitals and infrastructure, limited capacity, poor management, and unequal distribution are the major factors that affect availability (19–23). For cancer patients, timely and high-quality cancer care, treatment, therapy, and surgery are essential for a swift recovery; however, people should have good geographical accessibility, which is a significant barrier to accessing hospitals (24, 25).

Several studies in the Global South have shown that geographical proximity to hospitals is strongly associated with access to adequate healthcare (26, 27). According to the Nepal Living Standards Survey 2010-2011, only 61.8% of people could reach the nearest healthcare center within 30 min of travel time (28). Spatial accessibility modeling has been widely applied globally to identify service gaps, optimize facility locations, and inform equitable health resource allocation, particularly in low- and middle-income countries (29, 30). In Nepal, such studies are limited, focusing mainly on maternal health, immunization, and general hospital access (31, 32). These studies demonstrate that GIS-based accessibility analysis can reveal underserved populations, highlight transport barriers, and guide targeted interventions. Nepal has a diverse topography and population distribution, characterized by significant disparities between urban and rural areas. However, it has improved over the last two decades; 79.81% of people have access to primary health facilities using motorized transportation (33), highlighting an improvement in primary health accessibility.

Limited health infrastructure, geographic barriers, and uneven distribution of cancer care facilities exacerbate challenges in timely diagnosis and treatment. Previous initiatives, such as the National Cancer Control Program and selective establishment of oncology units in major hospitals, have made progress but remain insufficient to meet population needs, especially in remote and mountainous municipalities. These challenges highlight the urgent need for spatially informed planning and accessibility assessments to guide equitable allocation of cancer care resources across the country. Limited studies have been conducted regarding municipal-level spatial health studies in Nepal. It has varied the travel time to the nearest cancer hospital across different transportation modes and geographical regions within Bagmati Province. The study aims to evaluate the spatial accessibility of cancer hospitals in Bagmati Province, Nepal, under different travel scenarios and to identify geographic inequalities in access. The study hypothesized that the difference in travel time between walking and motorized scenarios is significantly smaller in urban municipalities than in rural mountainous ones. Considering the province’s diverse topography and unique population distribution, the study employed advanced geospatial applications to examine the accessibility and coverage of the population. Thus, the study explores the municipal-level spatial accessibility of cancer hospitals in Bagmati province. It estimates geographical accessibility and identifies accessible populations, thereby providing fact-based support for cancer care, prevention, and mitigation programs for stakeholders.

## 2 Methods and materials

### 2.1 Study area

**Figure 1:**
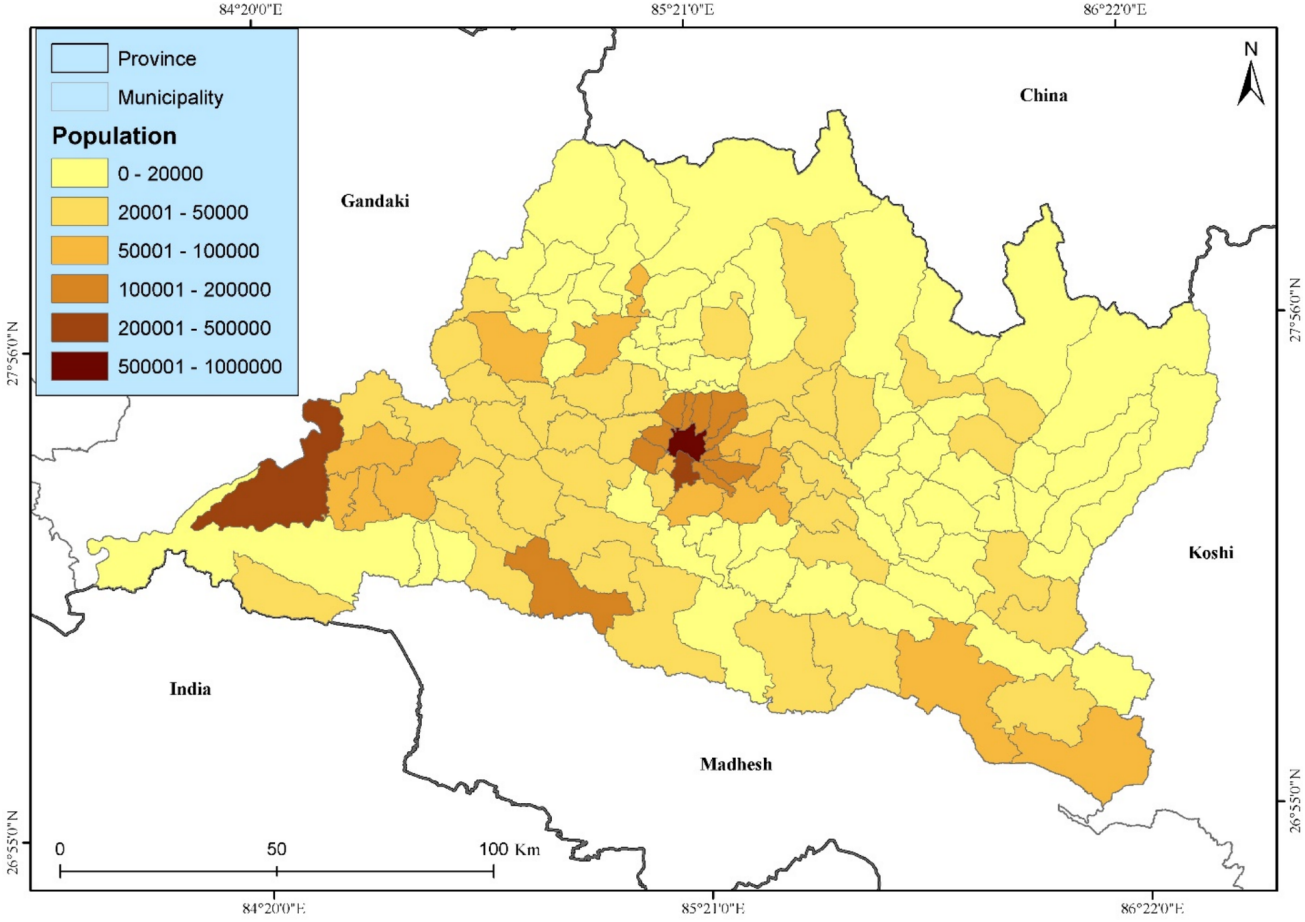
Location map of the study area with population distribution.

Bagmati Province is the most populated province of Nepal, located between 26°55′–28°23′N and 83°55′–86°34′E. It covers 13.79% of the country’s total area (Figure 1). It spans 20,300 square kilometers, with a forest cover of 27.29%. Administratively, it has 13 districts and 119 municipalities, including three metropolitan cities. Kathmandu Valley, the province’s urban center, is densely populated, housing approximately 3.1 million residents. It has a population of 5,433,818, comprising 2,761,224 females and 2,672,594 males (34). It has a well-developed road network, with four major highways (Mahendra, Prithibi, Araniko, and BP) connecting the Bagmati province; however, people living in the high-altitude regions (on the northern side of the province) of Rasuwa, Sindhupalchok, and Dolakha face difficulties accessing the road network. Agriculture is the basis of livelihood. Bagmati Province’s share of the national GDP is estimated to be 36.86 per cent this fiscal year. Agriculture and trade have the highest contribution.

Nepal’s health system is organized into federal, provincial, and local levels, with responsibilities distributed across the Ministry of Health and Population, provincial health directorates, and municipal health offices (35). Healthcare delivery includes primary health care centers, district hospitals, provincial hospitals, and specialized tertiary hospitals. In Bagmati Province, which encompasses the Kathmandu Valley and surrounding mountainous districts, health services are unevenly distributed. While urban municipalities such as Kathmandu, Lalitpur, and Bhaktapur have multiple tertiary hospitals with oncology services, rural and highland municipalities face limited service availability, long travel distances, and inadequate infrastructure. Bagmati Province has 35 public hospitals, including five dedicated cancer hospitals that provide comprehensive care, including screening, diagnosis, treatment, surgery, and palliative services. It has 41 Primary Health Care Centers (PHCCs) and 641 Health posts (36). Most hospitals are concentrated in the Kathmandu Valley, where they also offer hospice care, including counseling, health education, and psychosocial support. Despite their limited numbers, these hospitals play a crucial role in addressing cancer care needs throughout Bagmati Province.

The landscape of Bagmati Province is characterized by diverse topography, with over 75% of its area comprising hills and mountains (37). Elevations range from 141 meters above mean sea level (masl) in the southern plains of Tarai to 7,422 masl at Ganesh Himal in the north. The province is traversed by the Sunkoshi River, along with 10 sub-basins and 33 major rivers, making water resources a prominent feature of the region. Climatic conditions vary significantly, with annual precipitation ranging from 150 to 200 mm in the high Himalayas to 1,100 to 3,000 mm in the southern plains. Average annual temperatures range from 30 °C in the lowlands to −10 °C in the mountains, with most rainfall occurring during summer. Vegetation zones reflect this diversity, ranging from subtropical rainforests in the Tarai to alpine vegetation in the high-altitude regions. Additionally, socioeconomic conditions across the province vary widely, further highlighting its diversity and complexity.

### 2.2 Datasets

**Figure 2:**
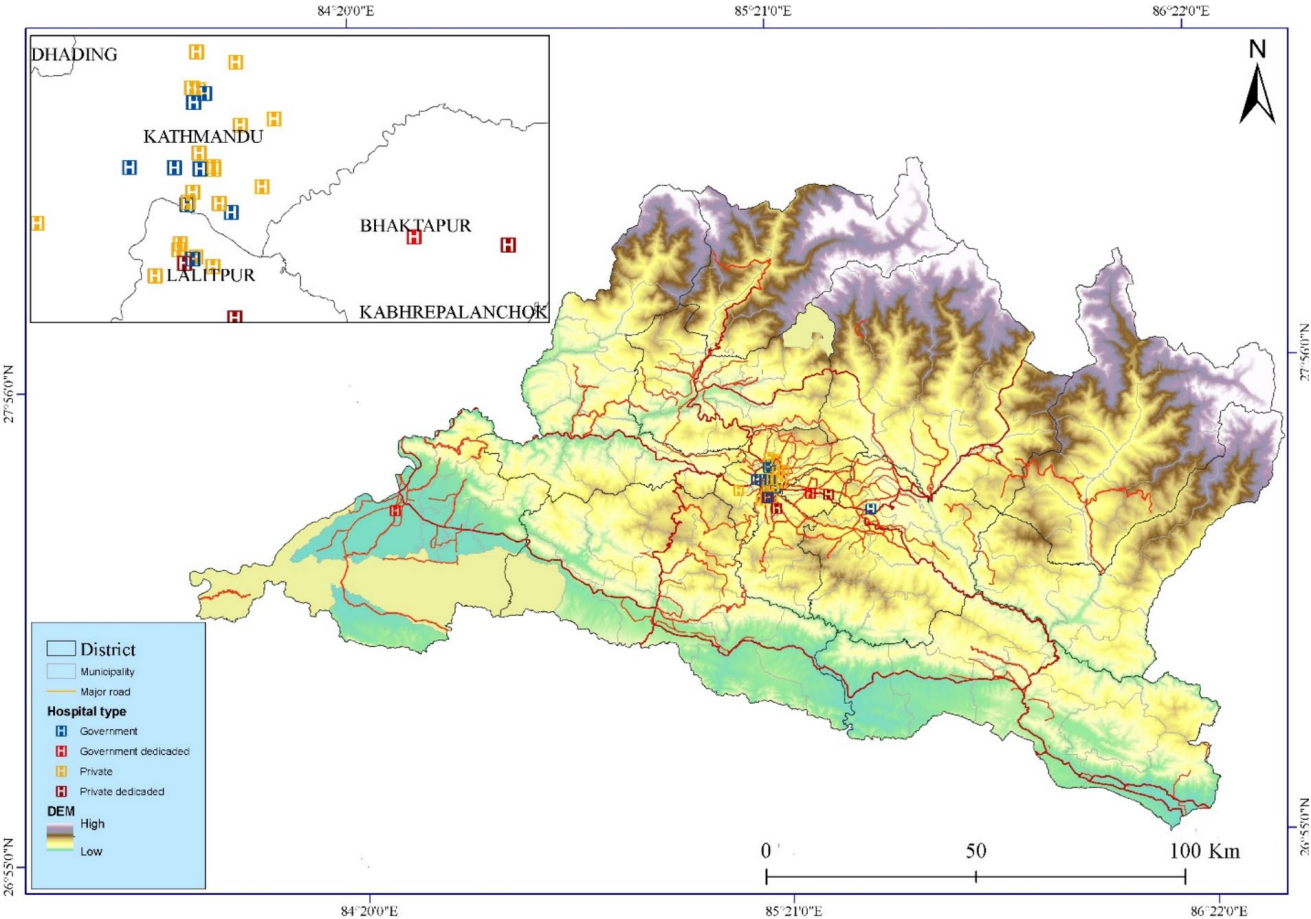
Spatial distribution of cancer hospitals and physical distribution of Bagmati province.

This study utilized two types of datasets: (i) spatial and (ii) non-spatial. Cancer hospital information was obtained from the Nepal Health Research Council (NHRC), using the latest report on cancer incidence and mortality rates in selected districts of Nepal (38). Additional details on hospital locations, types, services, and facilities. When assessing cancer hospitals, we integrated available indicators of facility functionality, including number of hospital beds, number of medical staff, ICU (intensive care unit) and CCU (cardiac care unit) availability, and the presence of diagnostic and therapy services. These attributes were used to differentiate the relative capacity and functionality of hospitals, providing a stratified understanding of service provision. It was collected through a detailed phone survey. Hospitals were categorized into four groups: dedicated government cancer hospitals, dedicated private cancer hospitals, government hospitals, and private hospitals (Figure 2). As of October 15, 2024, there were 2, 3, 9, and 19 hospitals in each group, respectively, providing cancer healthcare services. The coordinates of hospitals were determined using Google Earth. The study projected all data layers into the exact spatial resolution and reference frame. We used WGS84/UTM Zone 44N, and all raster data used in this study were adjusted to a 30-m resolution. All spatial datasets were projected to WGS84/UTM Zone 44N, which is suitable for Nepal’s longitudinal extent and provides minimal distortion in distance and area calculations within Bagmati Province. This projection ensures accurate travel-time and spatial accessibility analysis by preserving local metric properties, which are critical for cost-distance modeling in AccessMod.

Spatial data processing involved overlaying hospital locations on a land-use and land-cover (LULC) grid derived from Sentinel-2 satellite data. One hospital located on a major highway was manually adjusted to the nearest appropriate land-cover cell using Google Earth for accuracy. The geographical accessibility analysis employed geospatial technology and included steps such as collecting cancer hospital information, managing a GIS (geographic information system) database, modeling and analyzing data, and calculating results. Attributed datasets used in the analysis included population distribution, hospital types, available services and facilities, bed capacity, and healthcare staff numbers. Figure 2 visually represents the travel times to cancer hospitals across the study area, providing an overview of accessibility within Bagmati Province.

The study acquired municipal-level administrative boundaries from the Department of Survey, Government of Nepal. Elevation data was obtained from the Space Shuttle Radar Topography Mission (SRTM) digital elevation model (DEM) at a spatial resolution of 30 meters via the USGS Earth Explorer platform. The study area boundary for Bagmati Province was delineated using a spatial mask. Population data for 2020, with a spatial resolution of 1 km × 1 km, were sourced from the WorldPop dataset and adjusted to align with United Nations population estimates (33, 39). The gridded population map was used to analyze its relationship with land use land cover (LULC) data. LULC data were derived from ESA Sentinel-2 imagery at a 10-meter resolution using Google Earth Engine (GEE). The resolution of DEM was then increased to 30 m using the resampling technique of Cubic convolution interpolation.

Road network dataset, obtained from Open Street Map and further digitized using Google Earth, was reclassified into road classes encompassing: (1) primary roads, (2) secondary roads, (3) tertiary roads, (4) residential roads, and (5) paths. The data was rectified to connect segments of roads that were omitted during digitization and to delete those that extended into waterbodies. Using AccessMod, river, and OpenStreetMap (OSM) road network layers were aligned with the LULC data to match the resolution of the DEM and population grid. These layers were then merged to create a final land cover map (13).

To determine realistic travel times, the study considered the province’s existing road network, including road type, condition, and paths, as well as their suitability for both pedestrian and motorized access. We seek reputable services that calculate travel times based on real-life data, and the study validated their accuracy through discussions with local experts in the same field. Accessibility analysis, which estimated a low amount of travel time required for the population to reach the nearest cancer hospital, was conducted using AccessMod 5.0. Our study employed a least-cost path (friction surface) approach to generate a raster layer across the target area. Each gridded cell represented the minimum travel time from the cell’s location to the targeted destination. For this study, we set zero as the calculated maximum travel time to compute the travel time for the entire study area. The study reclassified each scenario-specific travel time raster layer and transformed it into three incremental travel time zones (within 30 min to more than 6 h) as polygon vectors. The population was redistributed onto a surface grid based on nine LULC classes and four road categories using ArcGIS (ESRI Inc., Redlands, United States, version 9.4). Zero population values were assigned to areas classified as water bodies.

### 2.3 Analysis

#### 2.3.1 Geographical accessibility analysis

AccessMod’s cost-distance modeling approach was adopted as the most appropriate method to estimate spatial accessibility to cancer hospitals in Nepal. This selection was guided by the limited availability of reliable health facility and patient flow data required for advanced methods such as the two-step floating catchment area (2SFCA) or gravity-based models. Given the fragmented nature of health information systems in Nepal, the cost-distance approach provides a practical and replicable solution for modeling travel time using topography, land use, road networks, and population distribution. It enables scenario-based comparisons across transportation modes (walking, cycling, and motorized) and effectively captures spatial inequalities in access under data-limited conditions. The least-cost path analysis was conducted in AccessMod to model travel time from each 100 m × 100 m grid cell to the nearest cancer hospital. The model integrates multiple spatial layers, including digital elevation model (DEM) for slope, land use/land cover (LULC), and road network data derived from OpenStreetMap and validated through manual digitization using Google Earth imagery.

The study aims to assess the potential spatial accessibility of the province’s total population to reach cancer hospitals for screening, diagnosis, and treatment purposes. In remote settings of the province, there are limited primary cancer screening services and referral mechanisms. The study calculated the travel time to the nearest cancer hospital to assess spatial accessibility in every gridded pixel within Bagmati Province. The study examines travel time under three scenarios: the motorized scenario, where people can access cancer hospitals via public transportation; the second cycling scenario, where people can access cancer hospitals by bicycle; and the third walking scenario, where people walk to the hospital. The road network was obtained from OpenStreetMap and supplemented through manual digitization using high-resolution Google Earth imagery to improve coverage and accuracy, especially in rural municipalities. Digitization followed a standardized protocol, including: (i) verification of existing OSM features; (ii) digitization of missing local and secondary roads; and (iii) topological correction to ensure network connectivity. The resulting network was validated through comparison with official transport maps and local administrative reports. We established different speed limits for various types of roads as recommended (16). LULC classes were assigned travel speeds based on a study by Huerta Munoz and Källestål (13), as shown in Table 1.

**Table 1:** Travel scenarios to the cancer hospitals.

Land cover type	Travel speed km/h					
	Walking	Bicycling		Motorized		
	Walking	Walking	Cycling	Walking	Cycling	Public transport
Water	0	0	0	0	0	0
Vegetation	1.5	1.5	0	1.5	0	0
Flood vegetation	2	2	0	2	0	0
Crops	1.4	1.4	0	1.4	0	0
Built-up	3	3	0	3	0	0
Bare land	2	2	0	2	0	0
Snow cap land	1	1	0	1	0	0
Rangeland	1.5	1.5	0	1.5	0	0
Path	3	0	5	0	5	0
Residential	3	0	5	0	5	0
Tertiary road	3	0	10	0	0	25
Secondary road	3	0	10	0	0	35
Primary road	3	0	10	0	0	45

**Figure 3:**
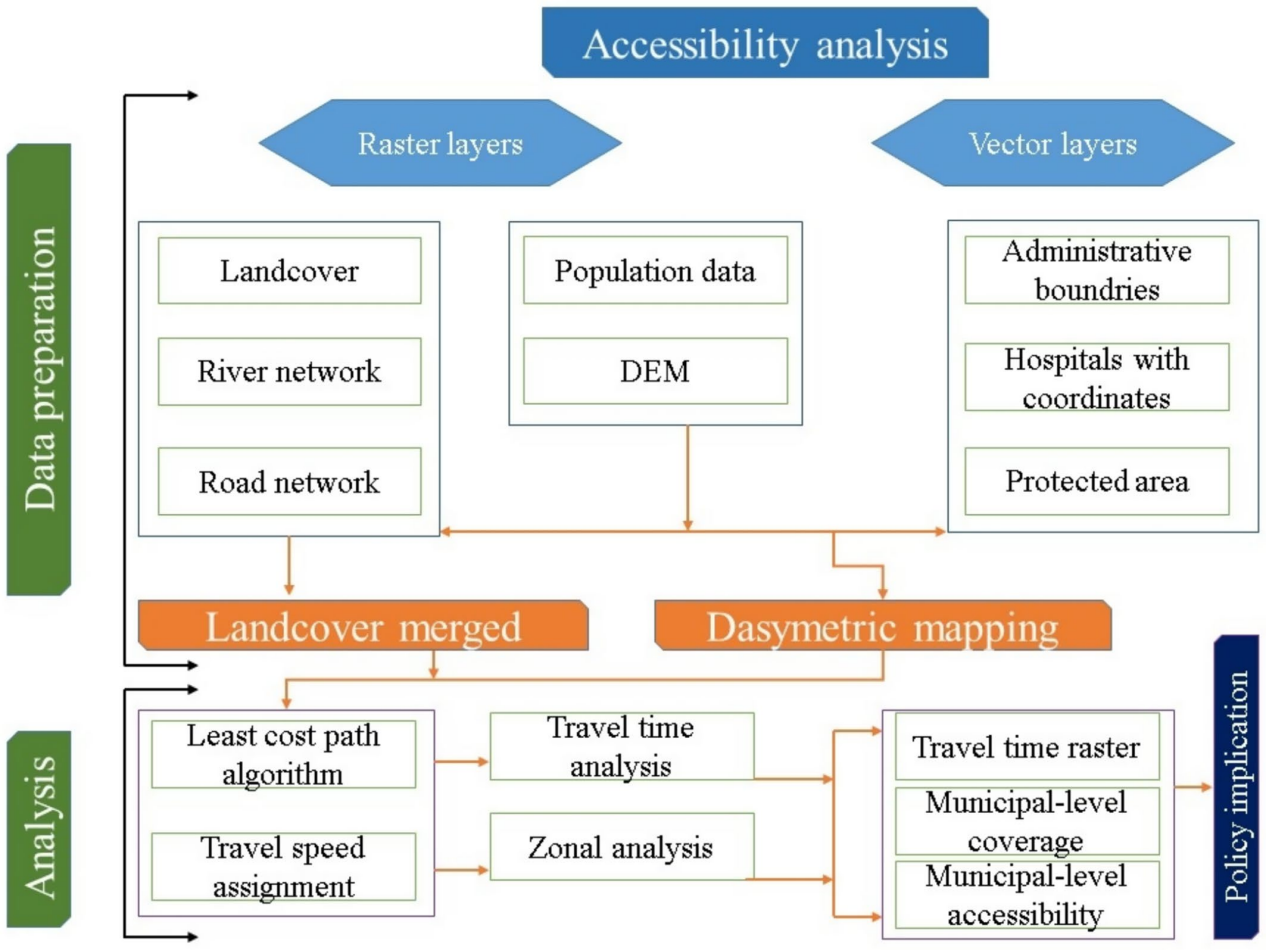
The overall flowchart of the geospatial approach assesses the geographical accessibility of the cancer hospital during the study.

During the analysis, we assumed that people used the shortest travel route to their destination, and we estimated the travel time from their residence to the cancer hospital using the least-cost path algorithm (40). Travel time was computed for the walking mode by substituting the friction pixel values for road travel and walking speed, based on land use and land cover. Furthermore, the study categorized the travel time into five groups (minutes): <0–30, 30–60, 60–120, 120–240, and <360, and summarized the municipal-level coverage of the population essential to each group (Supplementary material, Figures 1–3). The national average travel time has been calculated by adding the travel times of all gridded pixels. The respective population count weights each, and then this sum is divided by the total population. The study followed the overall actions for the accessibility analysis in Bagmati province (Figure 3).

Considering these three scenarios, the study utilized AccessMod 5.0 (WHO, Geneva, Switzerland) to calculate a raster surface of travel time between cancer hospitals and the population. The least-cost path algorithm method is employed to calculate the travel time. Water bodies have been considered barriers for cancer patients visiting the nearest cancer hospital. We set the zero speed for this particular land cover category as a barrier to prevent it from being used. We chose the anisotropic modes for the slope analysis, which explicitly takes into account slope derived from the DEM. AccessMod incorporated the DEM, including slope, into the analysis process due to the topographic variance in the Bagmati province. We adjust the travel speed based on uphill and downhill gradients, reflecting the impact of topography on travel scenarios. The topography and road network affect travel time, potentially increasing or decreasing speed in all travel scenarios.

## 3 Results

### 3.1 Geographical accessibility

**Figure 4:**
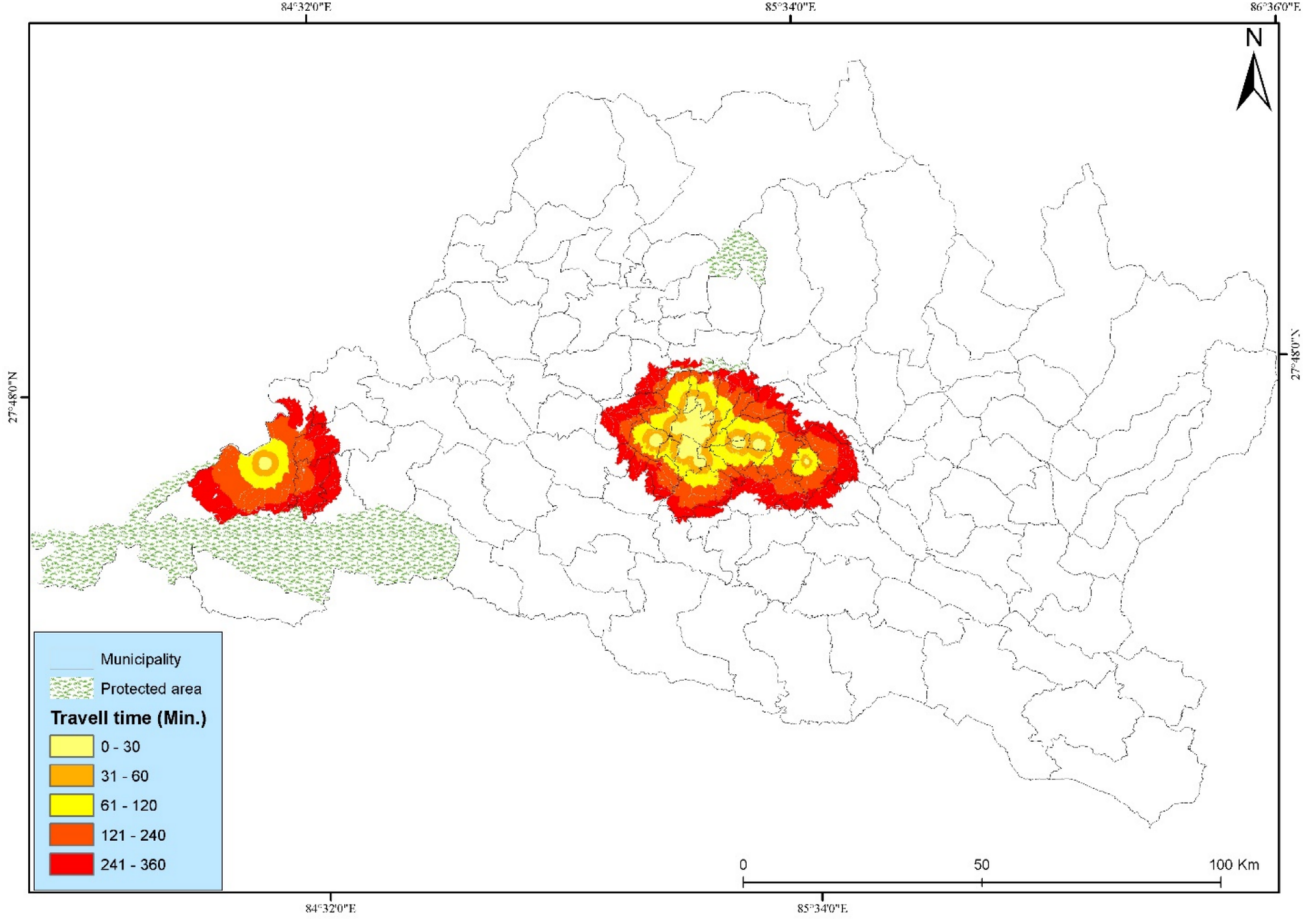
Spatial accessibility to cancer hospitals is based on a walking scenario.

**Figure 5:**
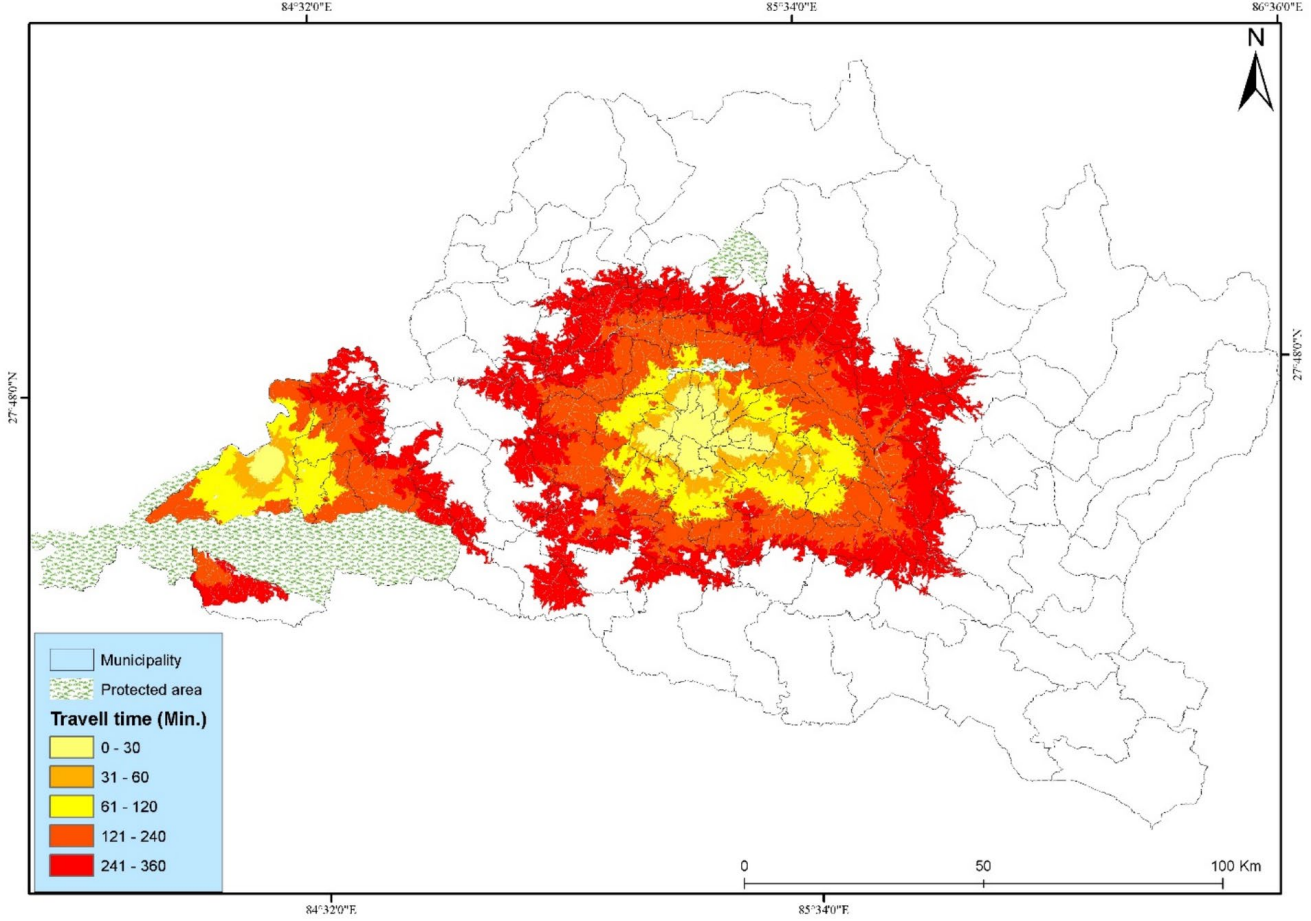
Spatial accessibility to cancer hospitals is based on a bicycling scenario.

**Figure 6:**
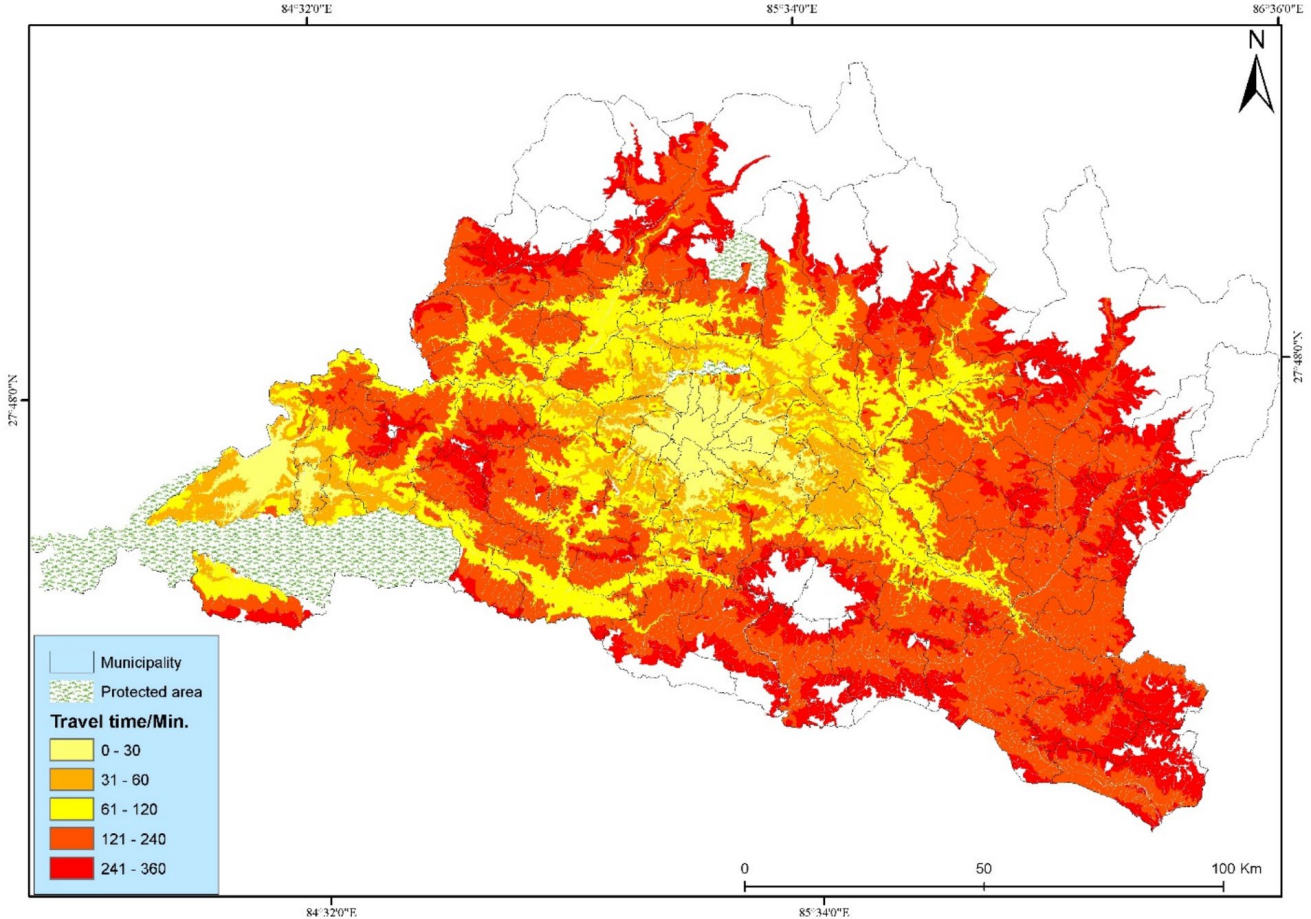
Spatial accessibility to cancer hospitals is based on a motorized scenario.

The travel time distribution of cancer hospitals and various travel time scenarios are presented in Table 2, and they are visualized in Figures 4–6. The spatial accessibility analysis revealed that people have the highest access through motorized scenario, followed by bicycling and walking (Table 2). The walking scenario has the lowest accessibility (39.76%); it covers only 15 municipalities within the 30-min travel time and around 104 municipalities, with 3,111,235 people without access to cancer hospitals. The study found that the motorized scenario has the maximum number of municipal coverage (114), followed by bicycle (86) and walking scenarios (35) within 360 min of travel time.

**Table 2:** Travel scenarios to the cancer hospitals in three different modes (walking, bicycling, and motorized).

Scenario/time	360 min	240 min	120 min	60 min	30 min
Motorized	96.78	94.28	85.76	80.54	72.57
Bicycling	86.43	80.97	76.2	71.1	65.62
Walking	76.51	74.14	69.55	60.8	39.76

The municipality-wise percentage of people served increases with faster means of transportation, with the motorized scenario having the highest percentage of access, followed by bicycles, and walking having the lowest access. The results show that the lowest spatial accessibility to cancer hospitals, based on the walking scenario, has been presented in (Figure 4). Road networks, public vehicles, and settlements are the major components that impact accessibility. The study found that most people have access within a six-hour travel time. In this study, cancer patients used Paths, Residential Areas, and roads, representing most of the road network in the urban and plain areas of Bagmati Province. Kathmandu Valley and Chitwan have good population coverage in walking scenarios. Most people are underserved in the walking scenario.

The study investigated the geographical accessibility and spatial coverage of existing cancer hospitals in the Bagmati Province, based on a maximum travel time of 360 min. Bicycling (bicycling) has improved walking accessibility, with 65.62% of the population covered (Figure 5). The details of the municipal-level population coverage list for the three scenarios are presented in Supplementary material. According to the results, 20 municipalities have access to bicycling scenarios, with 2,594,334 people within a 30-min travel time. It has improved coverage compared to the walking scenarios. Bicycling scenarios cover more than 86% of people within 6 hours of travel time. In bicycling, people are utilizing a bicycle after walking to the nearest road. It has higher coverage of the population being served.

The study found a significant increase in accessibility when people first walk to the nearest road network and then use public vehicles to reach the cancer hospitals. Motorized (motorized) has the highest accessibility, with 72.57% of people covered within the 30-min travel time (Figure 6). The motorized scenario has a maximum people coverage of 96.78%, and the walking scenario has a lower (76.51%) population coverage within 360 min of travel time. The use of a public vehicle along different road networks significantly decreases the travel time and distance within the fixed traveling time.

### 3.2 Municipality-wise accessibility

**Figure 7:**
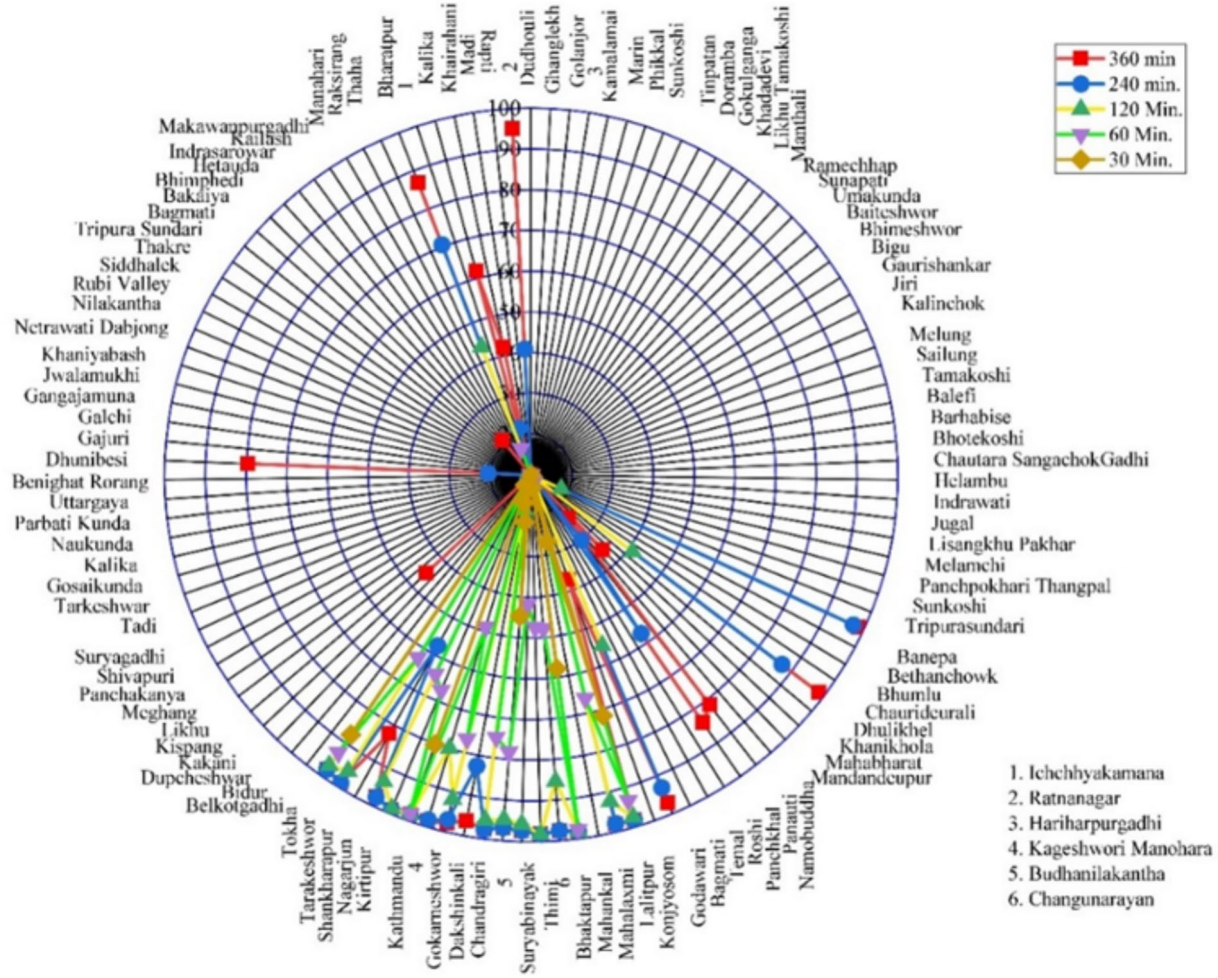
Radar charts illustrating variations in spatial accessibility indicators across municipalities in Bagmati province under different travel scenarios.

Under the walking scenario, the spatial accessibility analysis of cancer hospitals across Bagmati Province revealed notable disparities (Figure 7). Overall, 7 municipalities (5.3%) had no population coverage within the modeled walking travel time thresholds. An additional 77 municipalities (58.8%) demonstrated low accessibility, with 0–25% of their population covered. Moderate coverage levels were observed in 6 municipalities (4.6%), where 25–50% of residents had access by walking, and in 3 municipalities (2.3%) with 50–75% coverage. By contrast, high accessibility was achieved in 26 municipalities (19.8%), where 75–100% of the population could reach a cancer hospital on foot within the defined parameters. These results highlight significant spatial inequalities in walking accessibility to cancer care services across the province.

**Figure 8:**
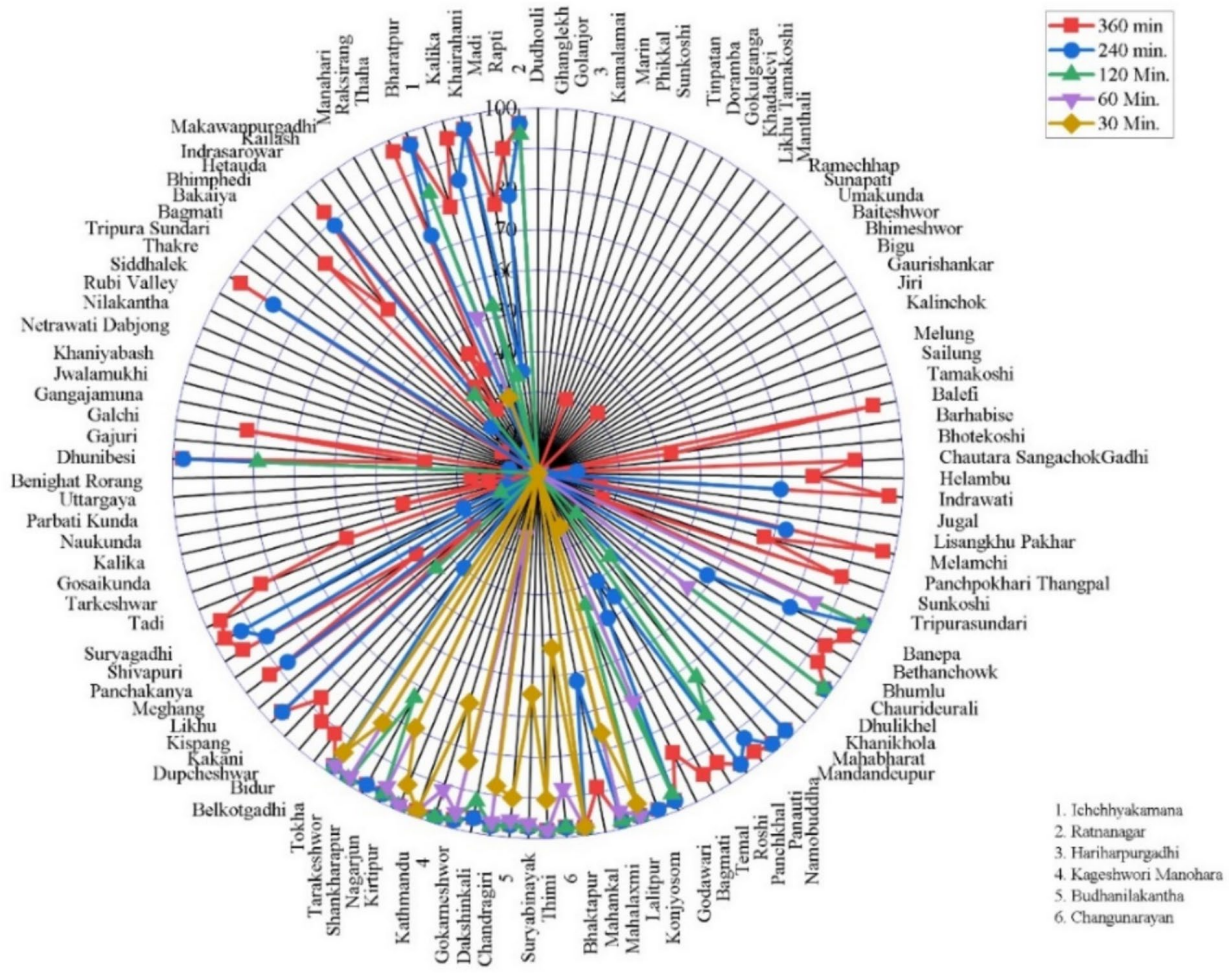
The spatial coverage of cancer hospitals in a bicycle scenario was presented in a radar chart.

In the bicycle scenario, the accessibility analysis of cancer hospitals in Bagmati Province demonstrated substantial improvements compared to walking access. However, it continued to exhibit uneven spatial distribution (Figure 8). A total of 7 municipalities (5.3%) remained without any population coverage within the bicycling travel time. Low coverage levels (0–25% of the population) were observed in 44 municipalities (33.6%). Moderate coverage was recorded in Manahari, Makawanpur Gadhi, and Umakunda municipalities (2.3%), with 25–50% coverage, and in 5 municipalities (3.8%), with 50–75% coverage. Notably, 60 municipalities (45.8%) achieved high accessibility, with 75–100% of their population able to reach a cancer hospital by bicycle. These results indicate that bicycling substantially expands spatial access to cancer care compared to walking, although important geographic disparities persist across the province.

**Figure 9:**
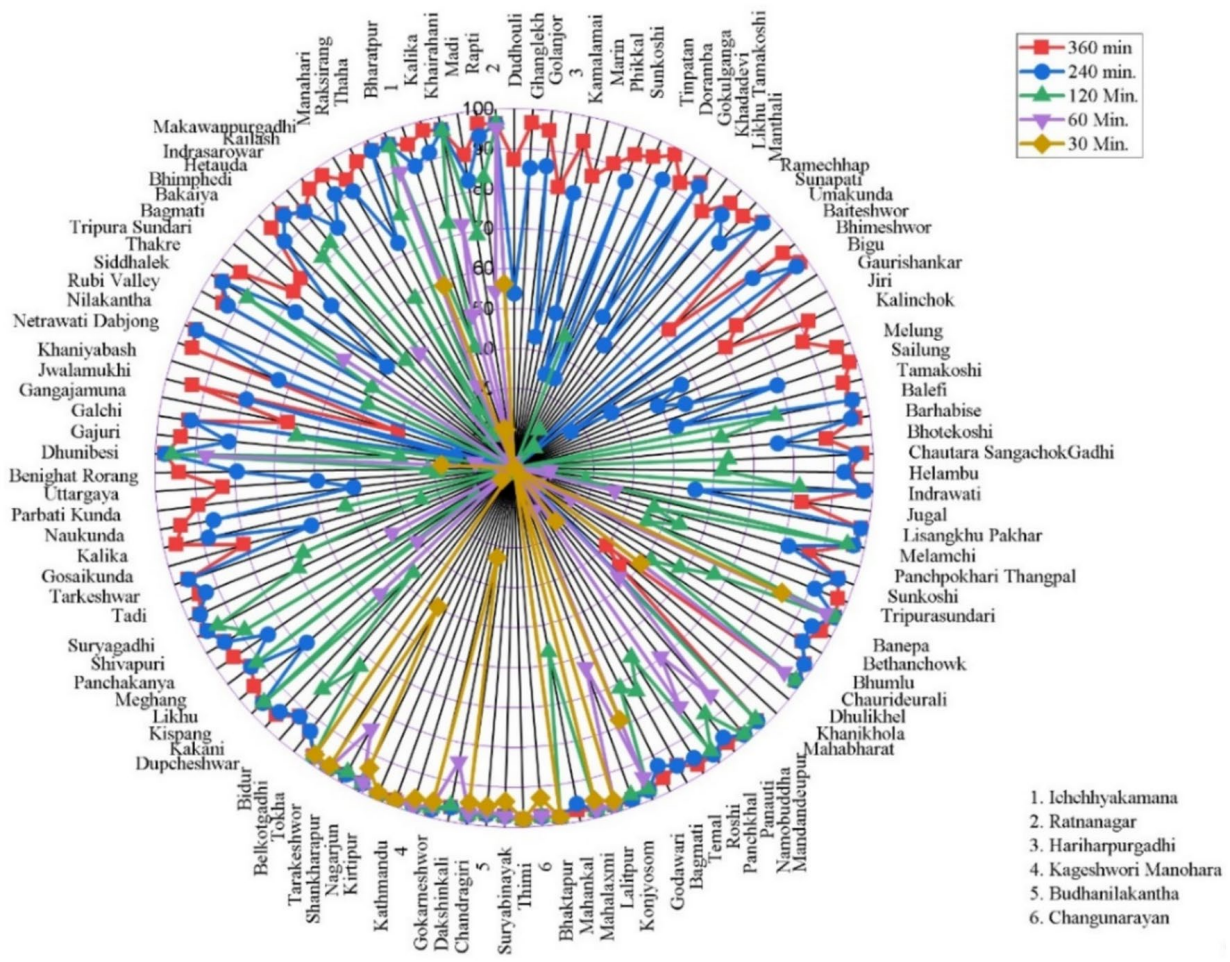
The spatial coverage of cancer hospitals in a motorized scenario.

Under the motorized travel scenario, the accessibility analysis of cancer hospitals in Bagmati Province demonstrated widespread and high coverage (Figure 9). The Ruby Valley rural municipality (0.8%) remained without any population coverage within the modeled motorized travel time thresholds. Low coverage levels (0–25% of the population) were observed in the Umakunda rural municipality (0.8%). Moderate access was found in Khaniyabas, Mahabharat, and Khanikhola municipalities (2.3%), with 25–50% coverage, and in Gangajamuna and Parbati Kunda municipalities (1.5%), with 50–75% coverage. Notably, 112 municipalities (85.5%) achieved high accessibility, with 75–100% of their population able to reach a cancer hospital by motorized transport within the specified travel time. These results indicate that motorized transportation provides the most extensive geographic access to cancer care services across the province.

## 4 Discussion

**Figure 10:**
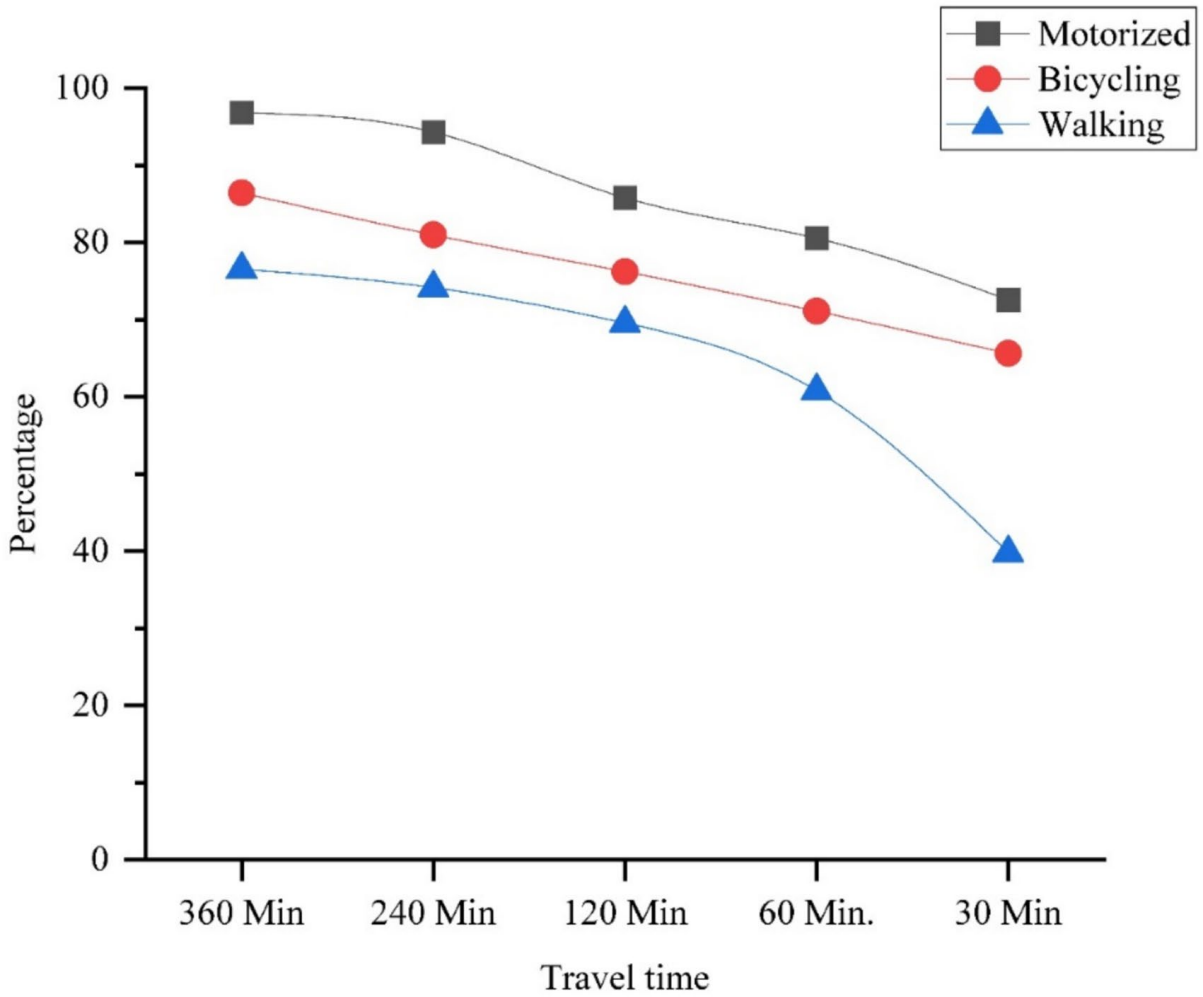
Covered population (%) by all three travel scenarios within the travel time in Bagmati province.

The study examined spatial accessibility in the Bagmati province based on a maximum traveling time of 360 min. Motorized scenario has the highest coverage among the three scenarios, being the best travel scenario, which covers 96.78% of the population (Figure 10). The study computed people’s travel times and found that there is high accessibility for the maximum number of people to the cancer hospital. However, we found a high level of spatial injustice in remote rural regions. Seventy-three municipality residents have zero accessibility within a 360-min travel time, as per walking scenario.

The study found that the Kathmandu Valley and Chitwan urban areas showed higher accessibility across all scenarios, which was attributed to their easy road networks. Based on the study, it was observed that all municipalities in the Kathmandu Valley and Bharatpur metropolitan cities have higher accessibility within all three scenarios. On the other hand, the municipalities in the hilly region of the province, particularly in the eastern and northern parts of Bagmati Province, were less accessible. Rural and mountainous regions in the middle hills of the northern and eastern parts of the province have low accessibility. The low accessibility in remote areas is attributed to steep slopes, scattered settlements, and sparse infrastructure. High-density areas, particularly in urban municipalities such as Kathmandu, Lalitpur, Bharatpur Metropolitan City, and Hetauda Sub-Metropolitan City, offer high accessibility within a short travel time.

Accessibility differs in three scenarios, primarily due to the lack of a well-developed road network, which hampers the provision of necessary health facilities to cancer patients in rural areas of the Bagmati province. Rubi Valley, Jugal, Bigu, Gaurishankar, Gosaikunda, and Umakunda are remote and hilly municipalities that require intervention to provide cancer treatment facilities. The study outlines the spatial coverage area of existing cancer hospitals in the Bagmati Province.

Additionally, it identifies major components that hinder the provision of adequate coverage of the population with crucial cancer treatment (41). Thus, the current study presents a more detailed and accurate examination than an approach that considers only one characteristic. The current study aims to contribute to a deeper understanding of the spatial aspects of cancer mitigation and identify potential gaps. Thus, the study’s findings may provide valuable insights for enhancing cancer mitigation programs and informing evidence-based planning and policy development.

The findings of this study align closely with ongoing health sector reforms and policy priorities in Nepal. Under the National Health Policy (2019) and the Federal Health System restructuring, provincial and local governments have been tasked with improving equitable access to essential health services. The National Cancer Control Strategy (2016–2020) and the Multisectoral Action Plan for the Prevention and Control of Non-Communicable Diseases (2021–2025) also emphasize early diagnosis, decentralized treatment, and the establishment of regional oncology centers. National projects such as the Strategic Road Network (SRN) expansion, provincial highway upgrades, and the Rural Access Program (RAP) are gradually enhancing connectivity across Bagmati Province, potentially reducing travel times to major hospitals. Furthermore, the integration of digital health systems, such as the Health Management Information System (HMIS) and emerging telemedicine platforms, is reshaping how patients in remote areas access specialist consultations.

The study examined how combining and analyzing spatial data using geospatial techniques can be integrated into the decision-making process for optimal resource allocation to mitigate the cancer burden in Bagmati Province. A significant disparity was observed between motorized and non-motorized modes of transportation in India (42). The study highlighted a significant variation between motorized and non-motorized scenarios, underscoring a moderate gap between bicycling and walking as modes of transportation. Similar results have been found in several research studies conducted in similar geographical settings and rural regions. This study highlights substantial spatial inequalities in access to cancer hospitals across Bagmati Province, with mountainous and remote municipalities such as Rubby valley, Kispang, Ghyanglekh, Temal, of Rasuwa, Nuwakot, Sindhuli, and Kavre of northern areas showing the longest travel times under all transportation scenarios. In contrast, municipalities within the Kathmandu Valley and major highway corridors exhibit significantly higher accessibility.

The study categorized travel time into five categories based on relevant cut-off points to summarize the population’s travel time, specifically 30 min, 60 min, 120 min, 240 min, and 360 min. Although much of the population is concentrated in the Kathmandu Valley and plain land (Chitawan), living in the urban area of Bagmati province can reach cancer hospitals in 30 min in all three scenarios; however, the people living in the rural municipalities in the northern and eastern sides of the province cannot reach the nearest cancer hospital within 360 min through the motorized mode. The study found that intra-municipality disparities are apparent in the study area, wherein backward and marginalized people, such as cancer patients living in rural municipalities with poor road networks, lack of hospitals, poor transportation facilities, and low economic resources, have to face ‘multiple risk and vulnerability (43, 44). The study assumed that the motorized travel scenario has access to public transportation on all road types, and it is frequent in urban areas, but less so in remote settings (45). Small vehicles, such as tempos, EVs, and minibusses, are popular in remote areas. People are using them as a local form of transportation, which provides easy and fast services, and they benefit from them in rural areas. They improve the accessibility for households located along minor roads without reliable public transport services. In Reality, people often must first walk to a location with limited road networks, and then catch public transportation on a main road.

The government of Nepal should establish cancer hospitals in remote and underserved areas. Mobile hospital services may help increase access and reduce spatial inequities in these rural areas. A study of geographical access to public health facilities in Nepal has found that 79.81% of the population has access to primary facilities within 30 min in walking mode; however, this is higher than the Nepal Living Standards Survey (33) indicating the geographical accessibility has improved over the last two decades. Motorized scenarios became the primary mode of travel for people seeking cancer hospitals. However, Nepal’s road network and public transport system are inferior (46, 47). The Nepalese government needs to increase its investment in improving the road network and public transportation system.

Several limitations should be acknowledged when examining and analyzing the study’s final results. The spatial analysis focused on the geographical accessibility of cancer hospitals in Bagmati Province, but it did not account for various determinants that could influence patients’ choices regarding treatment facilities, such as cost, income, culture, education, and awareness. These factors may significantly impact accessibility and should be taken into consideration in future research (48–50). Furthermore, the results of this study may not provide a comprehensive understanding of accessibility to cancer hospitals in Bagmati Province. While assessing geographical accessibility is a crucial aspect of overall accessibility, it is not the only factor to consider. AccessMod measured geographical accessibility solely based on travel time, without accounting for the hospitals’ efficiency and capacity, such as the number of doctors and healthcare staff, operating hours, available facilities, and bed capacity. Each hospital’s resources and capabilities vary, which in turn influences patients’ choices. Consequently, the limitations of this analysis may prevent a quantitative assessment of the number of cancer hospitals that individuals can access. Utilizing AccessMod, the analysis primarily focuses on the travel time required for individuals to reach the nearest cancer hospital, highlighting a significant aspect of the research.

This study acknowledges that the cost-distance approach does not explicitly incorporate facility capacity or supply–demand interactions. To partially address this, available indicators such as the number of hospital beds, medical staff, and availability of ICU, CCU, and therapy services were included to differentiate facility functionality. Future studies should integrate comprehensive datasets to apply 2SFCA or gravity-based models, enabling a more detailed understanding of the balance between healthcare supply and population demand in Nepal.

While this study primarily focused on the spatial dimension of accessibility, the broader 5A framework emphasizes that access to healthcare also depends on availability, affordability, accommodation, and acceptability. Due to data limitations, it was not possible to include these dimensions comprehensively. However, hospital-level indicators such as the number of beds, medical staff, and service availability were used to partially reflect availability. The study did not account for factors such as transport frequency, road quality, or patient preferences, which influence accommodation and acceptability. It does not account for social and economic factors, such as gender, income, or socioeconomic status, which can play an important role in healthcare access. Future research should integrate these additional dimensions using mixed-method or multi-criteria approaches to provide a more holistic understanding of access to cancer care in Nepal.

Although this study primarily focused on baseline accessibility conditions, the results provide a foundation for scenario-based planning applications in future research. The geospatial framework developed here can be extended to simulate potential interventions, such as the placement of new cancer hospitals in underserved municipalities, upgrading of key road networks, or seasonal accessibility adjustments that reflect monsoon-related disruptions. Incorporating such scenarios would enhance the decision-support value of the analysis and provide evidence for equitable health infrastructure planning. Future work will integrate these dynamic modeling components in collaboration with health authorities to guide strategic investment in cancer care services.

AccessMod calculates travel time based on the friction surface without accounting for seasonal variations. The road network in Bagmati Province is affected by rainfall. Nevertheless, seasonal impacts on road conditions are not factored in, which may compromise the accuracy of accessibility representation. The geographical accessibility assessed under the three travel scenarios (i.e., walking, bicycling, and motorized) provides insights into travel times across these modes. Furthermore, bicycles may not be a viable mode of transport in the hilly regions of the study area. Despite these limitations, the analysis identified municipalities with inadequate geographical accessibility, providing valuable insights for evidence-based public health planning, healthcare management, staffing and resource allocation for cancer mitigation in Bagmati Province. In the general case, people are seeking to travel less for diagnosis and treatment. Some cancer hospitals are providing services in neighboring provinces, but they are located far from the Bagmati province. Due to the long distance and longer travel times, people living in rural or peri-urban regions of the province require longer travel times to access hospitals within Bagmati Province than to access hospitals within the province itself. Although they exist with limited capacity compared to dedicated cancer hospitals, which provide services in Bagmati Province, and long distances mean that cross-provincial utilization is very low, the study was not included in the accessibility modeling. Future studies could further explore cross-provincial and cross-country care-seeking patterns to complement these findings.

## 5 Conclusion

The study examined the existing cancer hospitals at spatial scales, showing clear pathways to address and develop spatial health planning and appropriate policies. The study represents noticeable spatial differences in geographical accessibility and population coverage of the cancer hospitals across the three different travel scenarios. Additionally, the study demonstrates the utility of geospatial technology in leveraging combined datasets from multiple sources. It is beneficial in the Global South, where resources are limited. It helps to make evidence-based plans and decisions that support resource allocation. Nepal commits to achieving SDG 17 by 2030. Easy and equitable access to cancer hospitals is a significant task that the Nepalese government should ensure. Nepal is far from achieving its targeted goal; to reach this goal, the country needs to improve accessibility to hospitals. It is one of the significant tasks in achieving SDG-3.

It will provide evidence-based information that can be used to establish equitable cancer hospitals in the right locations, and it should also provide services in remote areas. More efforts should be focused on improving poor road networks in rural and remote areas, especially in the hilly regions of the northern and eastern parts of the country. The findings identify municipalities with high population but low accessibility, which should be prioritized for targeted interventions. These may include establishing mobile cancer screening or diagnostic units in underserved areas, upgrading key road segments to reduce travel time to major hospitals, and implementing telemedicine and digital health services to mitigate geographic barriers. Such measures can help improve equitable access to cancer care and guide evidence-based resource allocation in Bagmati Province.

## Data Availability

The raw data supporting the conclusions of this article will be made available by the authors, without undue reservation.
