# Supplementary material for: Geographical accessibility of cancer hospital using geospatial technology in Bagmati province, Nepal
Source: Front Public Health. 2026 Apr 17;14:1663989. doi: 10.3389/fpubh.2026.1663989 (PMC13133044; doi:10.3389/fpubh.2026.1663989)
Supplement: Supplementary file 1 [file Table_1.DOCX]

| Population coverage | Travelling scenario | | |
| --- | --- | --- | --- |
|  | Walking | Bicycling | Motorized |
| 0% | 7 | 7 | 1 |
| (0 - 25)% | 77 | 44 | 1 |
| (25 - 50)% | 6 | 3 | 3 |
| (50 - 75)% | 3 | 5 | 2 |
| (75 - 100)% | 26 | 60 | 112 |

|  | Walking | Bicycling | Motorized |
| --- | --- | --- | --- |
| 0% | Chaurideurali | Chaurideurali | Rubi Valley |
|  | Bagmati | Bagmati |  |
|  | Shivapuri | Shivapuri |  |
|  | Indrasarowar | Indrasarowar |  |
|  | Madi | Madi |  |
|  | Hariharpurgadhi | Hariharpurgadhi |  |
|  | Bigu | Bigu |  |
| <25% | Mahalaxmi | Lisangkhu Pakhar | Umakunda |
|  | Roshi | Khairahani |  |
|  | Manahari | Thaha |  |
|  | Lisangkhu Pakhar | Melamchi |  |
|  | Netrawati Dabjong | Jugal |  |
|  | Khairahani | Golanjor |  |
|  | Thaha | Panchpokhari Thangpal |  |
|  | Melamchi | Rapti |  |
|  | Jugal | Thakre |  |
|  | Lalitpur | Mahalaxmi |  |
|  | Golanjor | Indrawati |  |
|  | Indrawati | Temal |  |
|  | Ramechhap | Bhumlu |  |
|  | Nagarjun | Changunarayan |  |
|  | Panchpokhari Thangpal | Shankharapur |  |
|  | Bhimphedi | Raksirang |  |
|  | Rapti | Gokulganga |  |
|  | Namobuddha | Dudhouli |  |
|  | Thakre | Dakshinkali |  |
|  | Umakunda | Dhulikhel |  |
|  | Gokulganga | Ratnanagar |  |
|  | Temal | Phikkal |  |
|  | Bagmati | Mahabharat |  |
|  | Bhumlu | Sailung |  |
|  | Changunarayan | Kamalamai |  |
|  | Jwalamukhi | Bethanchowk |  |
|  | Konjyosom | Chautara SangachokGadhi | |
|  | Sunapati | Chandragiri |  |
|  | Shankharapur | Ramechhap |  |
|  | Panchkhal | Mandandeupur |  |
|  | Dakshinkali | Ghanglekh |  |
|  | Raksirang | Likhu Tamakoshi |  |
|  | Doramba | Banepa |  |
|  | Suryabinayak | Netrawati Dabjong |  |
|  | Dudhouli | Panauti |  |
|  | Bakaiya | Sunkoshi |  |
|  | Tamakoshi | Helambu |  |
|  | Ratnanagar | Kailash |  |
|  | Makawanpurgadhi | Manthali |  |
|  | Dhulikhel | Ichchhyakamana |  |
|  | Godawari | Tinpatan |  |
|  | Kispang | Sunkoshi |  |
|  | Likhu Tamakoshi | Benighat Rorang |  |
|  | Rubi Valley | Tripurasundari |  |
|  | Phikkal |  |  |
|  | Tinpatan |  |  |
|  | Mahabharat |  |  |
|  | Sailung |  |  |
|  | Kamalamai |  |  |
|  | Bethanchowk |  |  |
|  | Nilakantha |  |  |
|  | Khanikhola |  |  |
|  | Gangajamuna |  |  |
|  | Chautara SangachokGadhi | |  |
|  | Chandragiri |  |  |
|  | Bhimeshwor |  |  |
|  | Madhyapur Thimi |  |  |
|  | Benighat Rorang |  |  |
|  | Ichchhyakamana |  |  |
|  | Mandandeupur |  |  |
|  | Ghanglekh |  |  |
|  | Banepa |  |  |
|  | Kalika |  |  |
|  | Sunkoshi |  |  |
|  | Panauti |  |  |
|  | Baiteshwor |  |  |
|  | Helambu |  |  |
|  | Siddhalek |  |  |
|  | Kailash |  |  |
|  | Manthali |  |  |
|  | Tarakeshwor |  |  |
|  | Tokha |  |  |
|  | Kageshwori Manahora |  |  |
|  | Sunkoshi |  |  |
|  | Bhaktapur |  |  |
|  | Galchi |  |  |
|  | Tripurasundari |  |  |
| <50% | Tripura Sundari | Manahari | Khaniyabash |
|  | Balefi | Makawanpurgadhi | Khanikhola |
|  | Marin | Umakunda | Mahabharat |
|  | Jiri |  |  |
|  | Bidur |  |  |
|  | Gokarneshwor |  |  |
| <75% | Kirtipur | Bhimphedi | Gangajamuna |
|  | Khadadevi | Suryabinayak | Parbati Kunda |
|  | Bhotekoshi | Rubi Valley |  |
|  |  | Sunapati |  |
|  |  | Bhotekoshi |  |
| 100% | Khaniyabash | Lalitpur | Bigu |
|  | Hetauda | Khanikhola | Gaurishankar |
|  | Gaurishankar | Namobuddha | Hariharpurgadhi |
|  | Uttargaya | Bakaiya | Likhu Tamakoshi |
|  | Budhanilakantha | Tarakeshwor | Bagmati |
|  | Kalinchok | Baiteshwor | Madi |
|  | Melung | Konjyosom | Dudhouli |
|  | Barhabise | Kalika | Bakaiya |
|  | Mahankal | Doramba | Marin |
|  | Kathmandu | Panchkhal | Raksirang |
|  | Dupcheshwar | Tamakoshi | Panchpokhari Thangpal |
|  | Kakani | Kispang | Jugal |
|  | Panchakanya | Roshi | Netrawati Dabjong |
|  | Suryagadhi | Bagmati | Gosaikunda |
|  | Tadi | Galchi | Jiri |
|  | Tarkeshwar | Gangajamuna | Ichchhyakamana |
|  | Kalika | Bhaktapur | Ramechhap |
|  | Naukunda | Nagarjun | Gokulganga |
|  | Dhunibesi | Jwalamukhi | Bhotekoshi |
|  | Belkotgadhi | Tokha | Golanjor |
|  | Likhu | Marin | Gajuri |
|  | Bharatpur | Madhyapur Thimi | Kalika |
|  | Meghang | Balefi | Meghang |
|  | Gajuri | Kalinchok | Tinpatan |
|  | Gosaikunda | Jiri | Phikkal |
|  | Parbati Kunda | Nilakantha | Galchi |
|  |  | Gaurishankar | Benighat Rorang |
|  |  | Godawari | Makawanpurgadhi |
|  |  | Bhimeshwor | Manthali |
|  |  | Siddhalek | Bidur |
|  |  | Kageshwori Manahora | Rapti |
|  |  | Tripura Sundari | Kailash |
|  |  | Bidur | Sunkoshi |
|  |  | Gokarneshwor | Chautara SangachokGadhi |
|  |  | Kirtipur | Bharatpur |
|  |  | Khadadevi | Likhu |
|  |  | Khaniyabash | Dakshinkali |
|  |  | Hetauda | Belkotgadhi |
|  |  | Uttargaya | Hetauda |
|  |  | Budhanilakantha | Bhumlu |
|  |  | Melung | Chaurideurali |
|  |  | Barhabise | Bagmati |
|  |  | Mahankal | Shivapuri |
|  |  | Kathmandu | Indrasarowar |
|  |  | Dupcheshwar | Lisangkhu Pakhar |
|  |  | Kakani | Khairahani |
|  |  | Panchakanya | Thaha |
|  |  | Suryagadhi | Melamchi |
|  |  | Tadi | Thakre |
|  |  | Tarkeshwar | Mahalaxmi |
|  |  | Kalika | Indrawati |
|  |  | Naukunda | Temal |
|  |  | Dhunibesi | Changunarayan |
|  |  | Belkotgadhi | Shankharapur |
|  |  | Likhu | Dhulikhel |
|  |  | Bharatpur | Ratnanagar |
|  |  | Meghang | Sailung |
|  |  | Gajuri | Kamalamai |
|  |  | Gosaikunda | Bethanchowk |
|  |  | Parbati Kunda | Chandragiri |
|  |  |  | Mandandeupur |
|  |  |  | Ghanglekh |
|  |  |  | Banepa |
|  |  |  | Panauti |
|  |  |  | Sunkoshi |
|  |  |  | Helambu |
|  |  |  | Tripurasundari |
|  |  |  | Manahari |
|  |  |  | Bhimphedi |
|  |  |  | Suryabinayak |
|  |  |  | Sunapati |
|  |  |  | Lalitpur |
|  |  |  | Namobuddha |
|  |  |  | Tarakeshwor |
|  |  |  | Baiteshwor |
|  |  |  | Konjyosom |
|  |  |  | Doramba |
|  |  |  | Panchkhal |
|  |  |  | Tamakoshi |
|  |  |  | Kispang |
|  |  |  | Roshi |
|  |  |  | Bhaktapur |
|  |  |  | Nagarjun |
|  |  |  | Jwalamukhi |
|  |  |  | Tokha |
|  |  |  | Madhyapur Thimi |
|  |  |  | Balefi |
|  |  |  | Kalinchok |
|  |  |  | Nilakantha |
|  |  |  | Godawari |
|  |  |  | Bhimeshwor |
|  |  |  | Siddhalek |
|  |  |  | Kageshwori Manahora |
|  |  |  | Tripura Sundari |
|  |  |  | Gokarneshwor |
|  |  |  | Kirtipur |
|  |  |  | Khadadevi |
|  |  |  | Uttargaya |
|  |  |  | Budhanilakantha |
|  |  |  | Melung |
|  |  |  | Barhabise |
|  |  |  | Mahankal |
|  |  |  | Kathmandu |
|  |  |  | Dupcheshwar |
|  |  |  | Kakani |
|  |  |  | Panchakanya |
|  |  |  | Suryagadhi |
|  |  |  | Tadi |
|  |  |  | Tarkeshwar |
|  |  |  | Kalika |
|  |  |  | Naukunda |
|  |  |  | Dhunibesi |
